# General practice-based clinical trials in Germany - a problem analysis

**DOI:** 10.1186/1745-6215-13-205

**Published:** 2012-11-08

**Authors:** Eva Hummers-Pradier, Jutta Bleidorn, Guido Schmiemann, Stefanie Joos, Annette Becker, Attila Altiner, Jean-François Chenot, Martin Scherer

**Affiliations:** 1 Department of General Practice/Family Medicine, University Medical Centre Göttingen, Humboldtallee 38, 37073, Göttingen, Germany; 2 Institute of General Practice, Hannover Medical School, Carl-Neuberg Str. 1, 30625, Hannover, Germany; 3 Institute for Public Health and Nursing Research, Department for Health Services Research, University of Bremen, Grazer Str. 4, 28359, Bremen, Germany; 4 Department of General Practice and Health Services Research, University of Heidelberg, Voßstraße 2, 69115, Heidelberg, Germany; 5 Department of General Practice, Preventive and Rehabilitative Medicine, University of Marburg, Karl-von-Frisch Str. 4, 35032, Marburg, Germany; 6 Department of General Practice, University of Rostock, Doberaner Str. 142, 18057, Rostock, Germany; 7 Department of General Practice, Institute of Community Medicine, University of Greifswald, Ellernholzstr. 1-2, 17487, Greifswald, Germany; 8 Institute of Primary Medical Care, University Medical Centre Hamburg-Eppendorf, Martinistr. 52, 20246, Hamburg, Germany

**Keywords:** Clinical trials, General practice, Barriers, Comparative effectiveness research, Research support

## Abstract

**Background:**

In Germany, clinical trials and comparative effectiveness studies in primary care are still very rare, while their usefulness has been recognised in many other countries. A network of researchers from German academic general practice has explored the reasons for this discrepancy.

**Methods:**

Based on a comprehensive literature review and expert group discussions, problem analyses as well as structural and procedural prerequisites for a better implementation of clinical trials in German primary care are presented.

**Results:**

In Germany, basic biomedical science and technology is more reputed than clinical or health services research. Clinical trials are funded by industry or a single national programme, which is highly competitive, specialist-dominated, exclusive of pilot studies, and usually favours innovation rather than comparative effectiveness studies. Academic general practice is still not fully implemented, and existing departments are small. Most general practitioners (GPs) work in a market-based, competitive setting of small private practices, with a high case load. They have no protected time or funding for research, and mostly no research training or experience. Good Clinical Practice (GCP) training is compulsory for participation in clinical trials. The group defined three work packages to be addressed regarding clinical trials in German general practice: (1) problem analysis, and definition of (2) structural prerequisites and (3) procedural prerequisites. Structural prerequisites comprise specific support facilities for general practice-based research networks that could provide practices with a point of contact. Procedural prerequisites consist, for example, of a summary of specific relevant key measures, for example on a web platform. The platform should contain standard operating procedures (SOPs), templates, checklists and other supporting materials for researchers.

**Conclusion:**

All in all, our problem analyses revealed that a substantial number of barriers contribute to the low implementation of clinical research in German general practice. Some issues are deeply rooted in Germany’s market-based healthcare and academic systems and traditions. However, new developments may facilitate change: recent developments in the German research landscape are encouraging.

## Background

Research in general practice is fundamental to ensure patient safety and efficient patient care. Recently, there has been increasing awareness of the need for clinical trials in primary care settings, or extension of translational research projects into everyday primary care, which can provide evidence with significant impact on public health. Translating research ‘from the ivory tower to the village green’ (Chris van Weel) is essential to make research findings useful for the population, but requires sufficient infrastructure and funding opportunities [1-5]. In the US, major funding programmes have been launched to facilitate comparative effectiveness studies, many of which are expected to be primary care-based or to tackle public health needs, and translational research with an outreach into primary care [6-8]. An elaborate procedure of citizens’ conferences and discussion rounds decide the general agenda and those projects of utmost interest with maximal support and benefit of the public [6]. In the UK, primary care clinical trials in so-called practice-based research networks (PBRNs) are considered a priority within national funding programmes and a cornerstone for Britain’s economy [9]. Substantial efforts have been made to facilitate trials in general practice settings, including training and accreditation of ‘research ready’ practices, and there is a state-funded overhead coordination structure for the PRNBs [10,11]. In the Netherlands and the US, practice-based research networks provide continuous high-quality observational data for health services research, but are also expected to serve as a platform for clinical trials [12,13]. The UK-led European TRANSFoRm project aims to interlink and extend existing databases of electronic patient records, and to provide electronic support to practice networks in order to facilitate clinical trials and comparative effectiveness research in primary care [14,15]. Usually, practice-based research networks cooperate closely with specific coordinating centres, often academic departments for general practice, or specific, publicly funded research institutes for primary health care research.

In Germany, fundamental biomedical research and technological development are the strongholds of medical research. By comparison, clinical trials are still under-represented. This need has been recognised by stakeholders in research policy. To address this, a single national funding programme has been launched [16]. All clinical specialities (including general practice) can apply for funding in this highly competitive programme; the reviewer group is multidisciplinary and international. Similarly, a federal start-up funding programme has instigated a number of coordination centres for clinical trials, which are mostly based at universities/medical faculties. Interdisciplinary teams provide expertise and services related to the development of study protocols, administrative procedures and contract management, monitoring and biometry. After a few years of initial funding, however, these centres now have to be self-sustaining. Consequently, they rely heavily on industry-funded studies and investigator-initiated, low-budget trials are hampered. Moreover, the coordination centres are usually not familiar with the specific conditions and needs in primary care settings.

In Germany, clinical research has been almost exclusively based in university or tertiary care hospitals to date; clinical studies in general practice remain the exception [4,17-23].

### Network group

In 2009, a group of researchers from various academic departments of general practice interested and involved in clinical research applied to the German Research Foundation to fund a network. Their long-term aim is to facilitate the implementation of clinical trials in primary care in Germany, increasing the numbers of successful grant applications and execution of high-quality clinical trials. The network group defined three work packages to be addressed regarding clinical trials in German general practice: (1) problem analysis, and definition of (2) structural prerequisites and (3) procedural prerequisites. This paper presents the main results of the problem analysis and highlights the next steps for the network and future development of clinical research in German general practice generally.

## Methods

According to German Research Foundation rules, network size is limited to a maximum of 15 persons (half of whom must be junior researchers without a tenured position). Funding is intended to enable meetings of the group and to invite experts [24]. Structural support, staff, or funding for actual studies is not provided.

Participation in the network group was based on personal experience and expertise. The group has met face to face six times so far. In addition to the network members, external national or international experts (that is, on coordination of primary care-based research networks or biometry) were involved in some meetings (present during part of the meeting, or via telephone conference), or consulted individually by a member. The work packages (1) problem analysis, and definition of (2) structural prerequisites and (3) procedural prerequisites were addressed in an iterative discussion process based on the group’s own expertise and knowledge as well as a comprehensive review of published and German grey literature. The group identified problem areas, which were then looked at in more detail by small working groups. Research papers published by German general practice researchers were sought in Scopus (http://info.scopus.com) and by hand searching publication lists of general practice departments, details are given in [25]. Literature referring to prerequisites and circumstances specific to conducting clinical trials studies in primary care settings was retrieved in PubMed (2000 to 2012) searching for the MeSH term ‘Randomized Controlled Trials as Topic’ and keywords ‘primary care/general practice/family practice’ as well as ‘feasibility’, ‘recruitment’, and ‘design’. Titles and abstracts were screened to identify papers that addressed the methodological and circumstantial aspects of trial conduction and were not merely reporting clinical trial results. Additionally, reference lists of identified relevant papers were checked. Barriers and facilitators of successful trial conduction were then compared to the German setting. The working groups compiled preliminary results, which were then discussed face to face in the entire network group, and with the external experts when appropriate, until consensus was reached.

## Results

### Problem analysis

Against the background of the German research tradition and the general situation for clinical research outlined above, aspects of current funding policies, the situation of academic general practice as well as some features of the German health care system were identified as possible barriers to conducting clinical trials in German general practice.

### Funding of clinical research relevant to general practice

Obtaining funding for investigator-initiated clinical trials is challenging in Germany. Expenses for clinical trials are high due to high staff costs and extensive regulatory requirements. These are applied to any study investigating pharmacotherapy, including comparative studies of licensed drugs, and include the obligatory certified Good Clinical Practice (GCP) training for any trial physician/participating general practitioner (GP) (the length of training varies between four hours and several days, by region). Funding problems may be particularly acute for pragmatic studies or comparative effectiveness studies: the vast majority of clinical trials are funded by the pharmaceutical industry, either as commissioned contract research, or as investigator-initiated trials attractive to the industrial partner. They are usually run by highly specialised hospital-based clinicians. In contrast, research questions from a primary care perspective usually carry no substantial financial interest for industry partners as they often focus on the non-inferiority of less invasive and/or inexpensive interventions or comparison of established, off-patent therapies [2]. Examples could be the comparison of different analgesics or different exercise strategies for musculoskeletal pain; different antibiotics for male urinary tract infections; regular walks in prevention of falls in the elderly; stepping down/stopping of thyroxine treatment for chronic hypothyroidism/goitre; or diuretic treatment for chronic heart failure. Research in primary care almost exclusively depends on public funding. The federal Clinical Trials funding programme is highly competitive and the vast majority of reviewers are hospital-based specialists. Single-centre trials or pilot studies are not eligible for funding. Comparative assessment studies or interventions stepping down treatment tend to be classed as ‘not contributing to innovation or scientific advancement’. Funding of clinical trials through other channels is rare: the high costs of clinical trials (which include expenses for staff, drugs and service, monitoring and regulatory requirements) preclude funding by foundations or charities. Sickness funds invest in research only to a very limited degree and tend to limit projects to their own clients.

### Academic environment and situation of academic general practice

Germany lacks a long-standing tradition of primary care research [26,27]. Academic departments of general practice/family medicine or primary health care have been created comparatively recently (starting in the late 1970s), and are still not universal today. Currently, approximately two-thirds of the German medical schools have a full academic department of general practice, usually with a single professor and a few staff members. In many universities, academic GPs are not paid as physicians, but receive a considerably lower ‘researcher fee’ as they are not considered to directly contribute to patient care in the university hospital.

An overview of barriers for clinical trials initiated by academic general practice (and academic general practice research in general), identified within the structure and tradition of German universities and medical faculties, is shown below.

Problems and barriers at the university level in German general practice:

• Medical faculties

• Research profiles of medical faculties expected to focus on a few specific research fields: little awareness and little compatibility of general practice principles and research agenda with most universities’ current research priorities [28].

• University hospitals are aiming to be profitable, opening new ‘business areas’ in a market-oriented health care system. Primary care is not perceived as a profitable research field.

• University hospitals (and thus medical faculties) compete with general practitioners (GPs) for some aspects of (out-) patient care. Some are aiming to establish their own primary care facilities in competition with traditional practices, resulting in a conflict of interest for academic and collaborating GPs.

• Coordination centres for clinical trials need to fund themselves (after a publicly funded starting period of a few years). They usually have no experience with (research in) primary care. Good Clinical Practice (GCP) training is run as a self-funded (or commercial) activity, focused on hospital-based research.

• Universities/academic departments of general practice or the German College of General Practice/Family Medicine are not formally involved in vocational training of GPs, which is under the auspices of the regional chambers of physicians. Thus, research is not a part of vocational training, and awareness of primary care research as well as evidence-based medicine remains quite low [29]. One exception is the state of Baden Wurttemberg, where the regional government established a coordination centre for vocational training at an academic department of general practice.

• Academic departments of general practice

• Usually small, mostly ‘first generation’, without a long-standing research tradition

• Few trained general practice researchers (the overall number of GPs with a PhD degree is still less than 30)

• Limited experience in clinical trials

• No funding or (stable) support structure for research practices/practice-based research networks

Most academic departments collaborate with a number of general practices in their area, which are contracted to receive and teach undergraduate students. These teaching GPs are not considered university staff; they receive a small service-based fee for teaching but not for train-the-trainer activities. Training and supervision is usually provided by the local academic department of general practice (which in turn does not usually have a budget for this), and the German College of General Practitioners and Family Physicians. While many of these teaching GPs are willing to participate in research, there is no financial or organisational support for general practice-based research networks.

The small size of the academic departments makes clinical trials particularly challenging: due to a two-stage reviewing procedure in the federal funding programme, the time lag between submission of the initial and main proposals and the receipt of funding is at least a year. Study centre recruitment must be successfully completed before a proposal is considered in the second review round. This is very challenging in primary care, where study centres represent participating general practices run by individually motivated GPs. For most general practice-based studies, a relatively high number of practices/centres must be recruited to accommodate the relatively low incidence/prevalence of most conditions in a non-selected practice population. In order to conduct a clinical drug trial, all research staff as well as all participating GPs must be formally GCP trained by an accredited centre. Depending on the region of Germany, up to 16 hours of compulsory formal GCP training is required, constituting a considerable commitment for busy GPs. Another barrier is the high cost of the training, which adds to the trial costs.

### German health care system

Other structural barriers relate to the German health care system: there are no formal practice lists (as patients do not need to register with a practice), and GPs are not gatekeepers to specialist care [30] German GPs compete with other GPs and community-based specialists for patients, and manage a very high patient flow resulting in comparatively short consultation times [31,32]. This implies that it is almost impossible to define a reliable practice population/denominator, and that there are no clear borders between primary and secondary specialist care. Patients with a condition that qualifies them for trial participation may see a specialist in the first place (reducing the number of eligible patients in general practice), or in addition to seeing a GP. In this very market-oriented health system, many GPs are concerned about displeasing and losing patients. They tend to consider practice-based research as a potential conflict with, rather than contribution to, good patient care and service orientation [33]. Most general practitioners work in single-handed or small group private practices and employ a few ‘practice assistants’ who act as receptionists and fulfil some secretarial and medical tasks, but do not work as independently as a nurse or practice manager. Very few GPs have personal experience of research. Though many practices are fully computerised, there is a multitude of different practice software programmes, which provide neither support for trial or research documentation nor offer compatibility with other applications. All these factors - lack of research awareness and experience, lack of supportive facilities and technology, and a work environment perceived as competitive and high-pressure - are likely to hamper trial participation of GPs and patients [34].

### Structural prerequisites

Further to addressing the obstacles described above, facilitating clinical trials in German general practice will also require some fundamental change. The funding environment to support trials needs to be improved and should include provision for pragmatic or comparative effectiveness trials. This is a political goal, which cannot be achieved by a small group or within a short time.

Further implementation and upgrading of academic departments of general practice would enable the recruitment, training and retention of qualified researchers and research staff beyond single projects. This would facilitate proposals for and the conduction of clinical trials in primary care. Some structural or organisational support for general practice-based research networks could provide practices with a point of contact, information, training facilities as well as support with recruitment and inclusion phase of a trial. GPs and practice staff will need professional recognition and financial compensation for their training and the time invested in research. A very recent survey on attitudes of German GPs towards participation in clinical trials has been published elsewhere [35].

### Procedural prerequisites

A lot of support facilities and technology (including information technology), operating instructions and predefined standard procedures exist for clinical trials in hospital settings or specialised trial clinics. However, most are setting-specific and cannot be conveyed to the challenge of running a clinical trial in primary care simultaneously with patient care in a busy general practice [36]. Peer support as well as a database of general practice-specific research tools and standard operating procedures (SOPs) can help researchers, research practices and their teams to run clinical trials successfully, and to establish and disseminate specific expertise. A summary of specific relevant key measures, for example on a web platform, will be useful when planning and conducting studies in Germany. Examples include the incidence and prevalence of symptoms and diagnoses in German primary care, in order to calculate sample or effect sizes, pre-test probabilities or inter-cluster correlation coefficients. The platform should contain SOPs, templates, checklists and other supporting materials for researchers. Appropriate and affordable GCP training facilities for GPs, able to cater to the special requirements of trials in a general practice setting, are necessary, as well as research training facilities for practice staff. Training and material must be developed and accredited, or permitted for cooperative use with accredited coordination and training centres. Information technology and practice software which incorporates and supports recruitment and documentation for trials, that is, by implementing search programmes and electronic case report forms (CRFs), is substantial [14]. However, so far, German software vendors consider the marketing potential of such adaptations as insufficient to warrant them being a development priority.

## Discussion

Our problem analysis revealed that a substantial number of barriers contribute to the low implementation of clinical research in German general practice. Some issues are deeply rooted in Germany’s health care and academic systems and traditions. However, new developments may facilitate change: the 2010 modification to German drug law emphasises the need for comparative effectiveness studies: within three months the majority of newly licensed drugs must be assessed for superiority compared to the current standard treatment for the particular condition [37,38]. Pricing is then based on the results of this assessment. While this new legislation has the potential to boost comparative effectiveness studies, practical implications with regard to their organisation and funding still remain unclear.

Nevertheless, interest in general practice and general practice research is growing [39], fuelled by the need to provide health care to an ageing and increasingly multi-morbid population while containing costs. In the last 10 years, the German federal government has run a few successful programmes to build up research capacity in general practice. However, these have not been sustained. Nevertheless, with respect to health services research or quality improvement studies in particular, their structural [40] and scientific outputs [39,41] have been remarkable. A recent publication of the German Advisory Council for the Assessment of Developments in the Health Care System points out the importance of strong primary care [42]. The number of academic departments of general practice is slowly, but steadily increasing. Representation of GPs in decision-making and funding bodies is gradually improving: very recently, the German College of General Practitioners and Family Physicians was granted the right to propose candidates to the reviewer boards of the German Research Foundation and one candidate (EHP) was elected.

Some academic departments of general practice and participants of the network have published clinical studies that have received international attention [17,19,21,43]. The first general practice-based study funded by the clinical trials programme has recently started [44]. Despite the lack of funding or structural support, there are local initiatives to accredit research practices and create practice-based research networks [45]. The network group has conducted a survey, which indicates that a substantial proportion of teaching GPs and those attending continuous professional development programmes run by the college or academic departments declare themselves motivated to participate in clinical trials [34]. Within the context of one ongoing trial run by several members of the group, a concept of GCP training appropriate for general practice-based trials has been developed and piloted [36].

This problem analysis results from the work of a small network group with very limited, temporary funding. Our review of the international literature was conducted comprehensively and cannot be considered systematic, as relevant papers are not always easily identified due to often inconsistent or incomplete MeSH labeling [28]. However, the network group collates considerable expertise with members from most research-active departments or institutes of general practice in Germany, and a track record of own clinical research. While this small group cannot address structural barriers, it can provide some of the procedural prerequisites named above. A systematic review of general practice-based clinical studies has been conducted [25]. Key measures for general practice-based clinical trials as well as useful tools to prepare proposals and to conduct studies will be made available.

The first requirement of establishing inventories, providing materials, engaging practices interested in research and networking, and providing information and peer support has the potential to improve the quality and success of grant applications. The network group aims to further stimulate the development of additional research capacity and research strategies for clinical trials in general practice, and to be considered a research group eligible for (structural) funding by the German Research Foundation.

Ultimately, the network group aims to underpin cooperation with coordination centres and funding bodies by, for example, defining accreditation standards and rewards for participating practices. It could also act as a ‘clearing house’, reviewing study proposals and protocols, providing access to practices and facilitating bottom-up communication of research needs in primary care to funders and policy-making bodies [11].

## Conclusion

Successful conduction of clinical trials in general practice settings can be hampered by characteristics of a market-based health care system, insufficient research capacity and funding, and unfulfilled need for structural support and established facilitative procedures. Though we identified barriers related to the low implementation of clinical trials in Germany, many of these, as well as the prerequisites, may apply to other countries. Increasing recognition of the importance of primary care and its role in comparative effectiveness studies or translational research may facilitate new developments. In Germany, recent developments in the research landscape are encouraging.

## Abbreviations

CRFs: case report forms; GCP: Good Clinical Practice; GPs: general practitioners; PBRNs: practice-based research networks; SOPs: standard operating procedures; US: United States of America; UK: United Kingdom.

## Competing interests

The authors declare that they have no competing interests.

## Authors’ contributions

EH-P and JB initiated the network. EH-P is the network’s speaker, she wrote the article, with input from all other authors. JB wrote the funding proposal (with input from all other network members), she is the network manager. JB, GS, JFC and MS revised the draft on several occasions, AA, AB, and SJ contributed to the paper in several working sessions and correction rounds. All network members participated in the meetings and group discussions which provided the content for article. EH-P, JB and MS determined the final structure of the paper, all authors agreed to this version.

## Authors’ information

EHP, AA, JFC, MS, are professors of general practice and heads of the departments of general practice at their respective universities (Goettingen, Rostock, Greifswald, Hamburg ). AB is professor of general practice in Marburg. GS, JB, and SJ are general practitioners and senior research fellows. Further members of the network are Angela Buchholz (senior research fellow, psychologist, Hamburg), Ildikó Gágyor (senior research fellow, GP, Göttingen), Jörg Haasenritter (senior research fellow, nursing scientist, Marburg) Frank Peters-Klimm (senior research fellow, GP, Heidelberg), Michael M Kochen (retired professor of general practice and past president of the German College of General Practitioners and Family Physicians), Antonius Schneider (professor of general practice, head of the institute of general practice, Technical University Munich) and Wilhelm Niebling (professor of general practice, head of the teaching department of general practice, University of Freiburg).
